# Letter to the Editor: “Lavender Products Associated With Premature Thelarche and Prepubertal Gynecomastia: Case Reports and Endocrine-Disrupting Chemical Activities”

**DOI:** 10.1210/clinem/dgaa226

**Published:** 2020-05-07

**Authors:** Jean-Marc Giroux, Marie Orjubin

**Affiliations:** 1 Consortium HE coordinator, Consortium Huiles Essentielles (Consortium HE), Aix-en-Provence, France; 2 Consortium HE Project Manager, Consortium Huiles Essentielles (Consortium HE), Aix-en-Provence, France

Dear Sir:

We read with great interest the paper by Ramsey et al (1), however we have some concerns with their conclusions and would like to point out some inconsistencies between the Ramsey et al statements and the products under investigation: Crusellas Violet Water Cologne, Mi Tesoro Agua de Violetas, and Baby Magic Calming Baby Bath. All 3 products are presented as containing lavender essential oil, which leads to cases of gynecomastia.

We carefully analyzed by gas chromatography–mass spectrometry and liquid chromatography with tandem mass spectrometry the Crusellas Violet Water Cologne and the Baby Magic Calming Baby Bath product mentioned in the Ramsey et al paper. In fact, as expected, after our review of the composition list and confirmation by analysis, we determined the Crusellas Agua de Violetas Cologne does not contain essential oil or natural fragrances. This paper spotlights the lavender essential oil, which is not present in the product. Instead, the following synthetic compounds were found: fragrances with an allergenic potential such as alpha-isomethyl ionone (2); dyes such as azo dyes, which are toxicologically suspected (3); and diethyl phthalate, suspected to cause endocrine disruption (4).

In regard to the Baby Magic Calming Baby Bath, the content of lavender oil is very low. The presence of other molecules or contaminants that could contribute to premature thelarche and prepubertal gynecomastia should be investigated, especially since the in vitro study methodology is questionable. Indeed, a very high amount of the individual oil’s components (a million times higher than the estradiol levels) is necessary to obtain a weak activity compared with the activity obtained from the whole essential oils (8-fold vs 50-fold increase) (1), and the compound's bioavailability was not taken into account.

Moreover, the authors raised relevant questions regarding a dissolution of bisphenols or phthalates from the plastic assay plates by the essential oils. However, to address this concern, they ran comparison tests with corn and soybean oil, which are edible oils. Vegetable oils do not have the same composition or properties as essential oils, which are known to be incompatible with the plastic that they dissolve (5). Thus, this test is not appropriate, and the in vitro results on the essential oils may have been due to contamination by endocrine-disrupting chemicals from the plastic labware (6).

Therefore, because the product used in this study in terms of composition, bioavailability, and contamination had not been correctly analyzed, the conclusion “LO (lavender oil) and TTO (tea tree oil) possess EDC (endocrine-disrupting chemical) activities that should be considered in the evaluation of premature breast development in girls and gynecomastia in boys and adult men” is highly questionable. In conclusion, no causal link can be established between the cases of gynecomastia reported by Ramsey et al and lavender essential oil.

## Additional Information

***Disclosure Summary***: The Consortium Huiles Essentielles represents the common interests of 11 French essential oil companies and has funded the authors. J.M.G. coordinates the Consortium Huiles Essentielles and is chairman of COSMED. M.O. is employed by the Consortium Huiles Essentielles.
